# Effects of the walking independence on lower extremity and trunk muscle activity during straight-leg raising following incomplete cervical cord injury

**DOI:** 10.1038/s41598-024-55039-z

**Published:** 2024-02-22

**Authors:** Tatsuya Sugimoto, Ryoto Yoshikura, Toshiyuki Maezawa, Kojiro Mekata, Yuya Ueda, Hiroshi Kawaguchi, Shintaro Izumi

**Affiliations:** 1 Department of Rehabilitation, Japanese Red Cross Kobe Hospital, Kobe, Japan; 2 Graduate School of System Informatics, Kobe University, Kobe, Japan; 3 Graduate School of Science Technology and Innovation, Kobe University, Kobe, Japan; 4 Shijonawate Gakuen University Faculty of Rehabilitation, Osaka, Japan; 5 Kobe University Graduate School of Health Sciences, Kobe, Japan; 6 Osaka Heat Cool Inc., Osaka, Japan

**Keywords:** Medical research, Health care

## Abstract

The purpose of this study was to compare the acceleration and surface electromyography (EMG) of the lower extremity and trunk muscles during straight-leg raising (SLR) in patients with incomplete cervical cord injury according to their levels of walking independence. Twenty-four patients were measured acceleration and EMG during SLR held for 10 s. Data were analyzed separately for the dominant and nondominant sides and compared between the nonindependent (NI) and independent (ID) groups based on their levels of walking independence. Frequency analysis of the EMG showed that the high-frequency (HF) band of the contralateral biceps femoris (BF) in the ID group and bands below the medium-frequency (MF) of the BF and the HF and MF bands of the rectus abdominis in the NI group were significantly higher during dominant and nondominant SLR. During the nondominant SLR, the low-frequency band of the internal oblique and the MF band of the external oblique were significantly higher in the NI group. The ID group mobilized muscle fiber type 2 of the BF, whereas the NI group mobilized type 1 of the BF and types 2 and 1 of the trunk muscles to stabilize the pelvis. This result was more pronounced during the nondominant SLR.

## Introduction

In Japan, the rate of incomplete cervical cord injury (ICCI) has been recently increasing owing to an aging population and changes in injury mechanisms, resulting in an increased rate of falls and stumbles^1^. Because patients with ICCI have varying degrees of motor and sensory impairment and improvement, we discuss whether patients can eventually walk independently in terms of goal setting. Previous studies reported age^2^ and American Spinal Injury Association (ASIA) lower-extremity motor score (LEMS)^3,4^ as factors associated with walking independently. However, there were cases in clinical practice showing a discrepancy between the improvement in LEMS and their level of walking independence, suggesting a decline in trunk muscle function. In fact, there are several studies that have investigated the association between trunk function and walking ability in patients with spinal cord injuries^5–7^, emphasizing the significance of trunk function for movement performance. Therefore, we believe that evaluating both limb and trunk motor function is necessary to predict functional prognosis.

We focused on straight-leg raising (SLR) exercise as a combined functional assessment of the lower limb and trunk muscles. Previous studies measuring surface electromyography (EMG) during SLR in healthy subjects reported the importance of pelvic girdle stabilization through the activity of the contralateral biceps femoris (BF) and bilateral trunk muscles, such as the psoas major, internal oblique (IO), and transversus abdominis, to counteract the anterior pelvic tilt associated with hip flexion of the rectus femoris (RF)^8–13^. This is because the rectus femoris, biceps femoris, and trunk muscles are attached to either the iliac, sciatic, pubic, or sacral components of the pelvis, and they act to tilt the pelvis anteriorly or posteriorly and contribute to pelvic stabilization^14–17^. Conversely, previous studies comparing EMG during SLR in healthy subjects and patients with chronic pain reported increased muscle activity in the ipsilateral RF, IO, and external oblique (EO)^18,19^, decreased EMG activity in the contralateral IO^9^, and delayed onset time of EMG activity in the ipsilateral IO^20^. Thus, these findings may be more evident when evaluating EMG during SLR in patients with ICCI who have generalized motor dysfunction.

We hypothesized that SLR would be a useful tool for assessing lower extremity and trunk muscle function in predicting functional prognosis in patients with ICCI. Therefore, this study aims to analyze the acceleration and muscle activity measured in the lower extremity and trunk during SLR in patients with ICCI and compare them according to their levels of walking independence upon discharge or transfer from an acute care hospital.

## Results

Twenty-four patients were analyzed: 8 in the NI group and 16 in the ID group. One patient from each group had missing LEMS data, so the remaining 7 in the NI group and 15 in the ID group were compared. In terms of basic information shown in Table 1, LEMS was significantly higher in the ID group than in the NI group (P = 0.00391). The Spinal Cord Independence Measure (SCIM) score for item 12 (“Mobility Indoors”)^21^ was significantly higher in the ID group than NI group at the time of SLR measurement and discharge or transfer from our hospital (both P < 0.001). Other walking indicators are shown in Supplementary Table S1 online.

**Table 1:** Basic information for each group (M: male, F: female).

	NI (n = 8)	ID (n = 16)	P values
Age (years)	70.9 (13.7)	65.2 (14.1)	0.35768
Gender	7 M, 1 F	13 M, 3 F	1
Height (cm)	162.3 (8.1)	162.2 (8.5)	0.96551
Weight (kg)	65.4 (11.4)	63.2 (14.1)	0.69198
SLR acquisition since injury (days)	6.5 (11.4)	6.2 (4.4)	0.95908
SLR measurement since injury (days)	14.6 (7.7)	13.3 (4.6)	0.65131
Independent walking since injury (days)	None	10.1 (4.8)	None
Total length of stay (days)	26.8 (16.2)	22.2 (6.8)	0.46601
LEMS (from 0 to 50 points)	41.1 (5.9)	48.5 (3.0)	**0.00391**
SCIM score for “mobility indoors”			
Initial evaluation (from 0 to 8 points)	0 (0)	4.2 (3.4)	None
SLR measurement	0.8 (1.4)	6.7 (2.1)	** < 0.001**
Discharge/transfer	1.5 (1.6)	7.6 (1.1)	** < 0.001**

One patient from each group had missing LEMS data. Data are shown as mean (standard deviation) except for gender. P values less than 0.05 are highlighted in bold.

### Acceleration variables (Supplementary Fig. S1 online)

The values for the lower leg were the highest on both sides. The root mean square (RMS) of the X-axis (anteroposterior direction) in the lower leg during the nondominant SLR showed a significant interaction (P = 0.0299) and was significantly higher in the NI group than in the ID group only at 5 s (P = 0.0048).

### EMG onset time (Supplementary Table S2 online)

The onset times for the RF and BF were calculated for all trials. The trunk muscles were averaged, except for trials in which they could not be calculated; patients whose onset times could not be calculated in all three trials per leg were excluded. Overall, RF and BF were activated before the start of the SLR, and the trunk muscles were subsequently activated. The onset time of the contralateral IO during the nondominant SLR was significantly lower in the NI group than in the ID group (P = 0.01521).

### EMG amplitude (Figs. 1 and 2)

**Figure 1:**
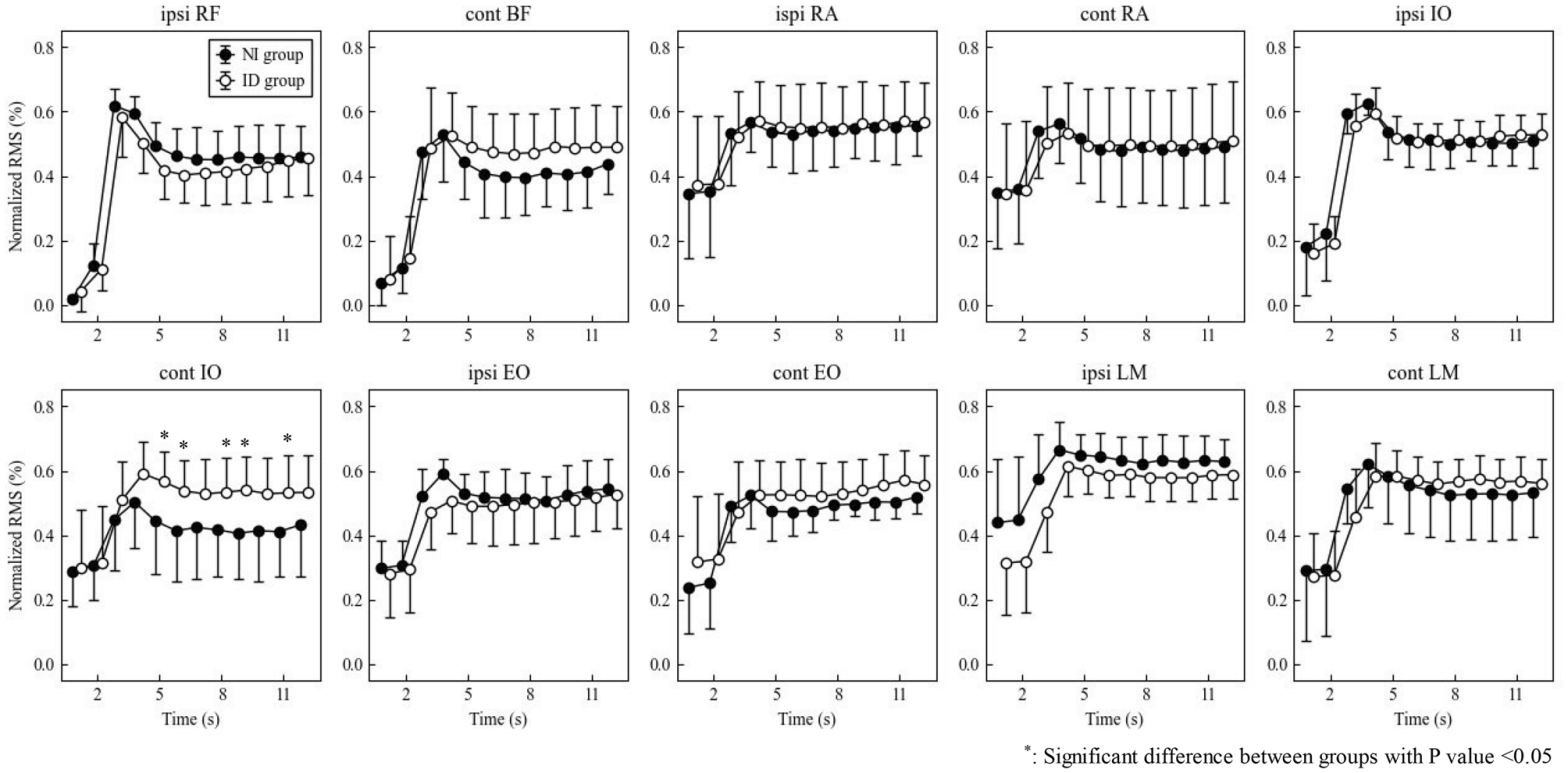
EMG amplitude RMS during dominant SLR. Contralateral IO was significantly higher in the ID group at 5, 6, 8, 9, and 11 s (all P < 0.05).

**Figure 2:**
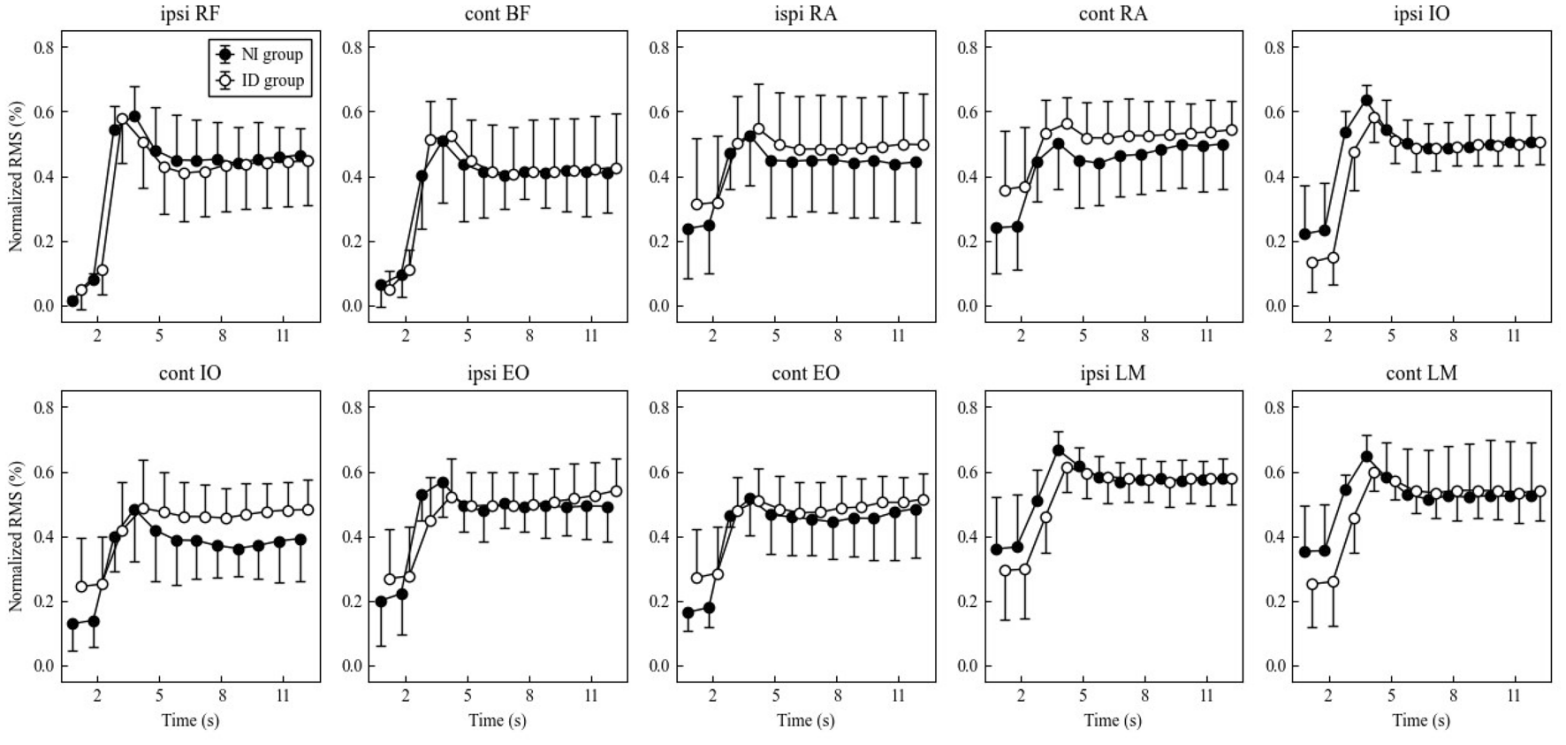
EMG amplitude RMS during nondominant SLR. There were no significant between-group main effects and interactions.

On both sides, all muscles peaked at 3 to 4 s (1 to 2 s starting SLR) and remained between 0.4 and 0.6 thereafter. The degree of increase from rest was the greatest for RF, followed by BF and ipsilateral IO. IO and EO during SLR were greater on the ipsilateral side than on the contralateral side.

Contralateral IO during the dominant SLR showed a significant interaction (P = 0.0044) and was significantly higher in the ID group than in the NI group at 5, 6, 8, 9, and 11 s (P = 0.0297, 0.0272, 0.0337, 0.0150 and 0.0350, respectively).

### Frequency analysis (Figs. 3, 4 and Supplementary Fig. S2 online)

**Figure 3:**
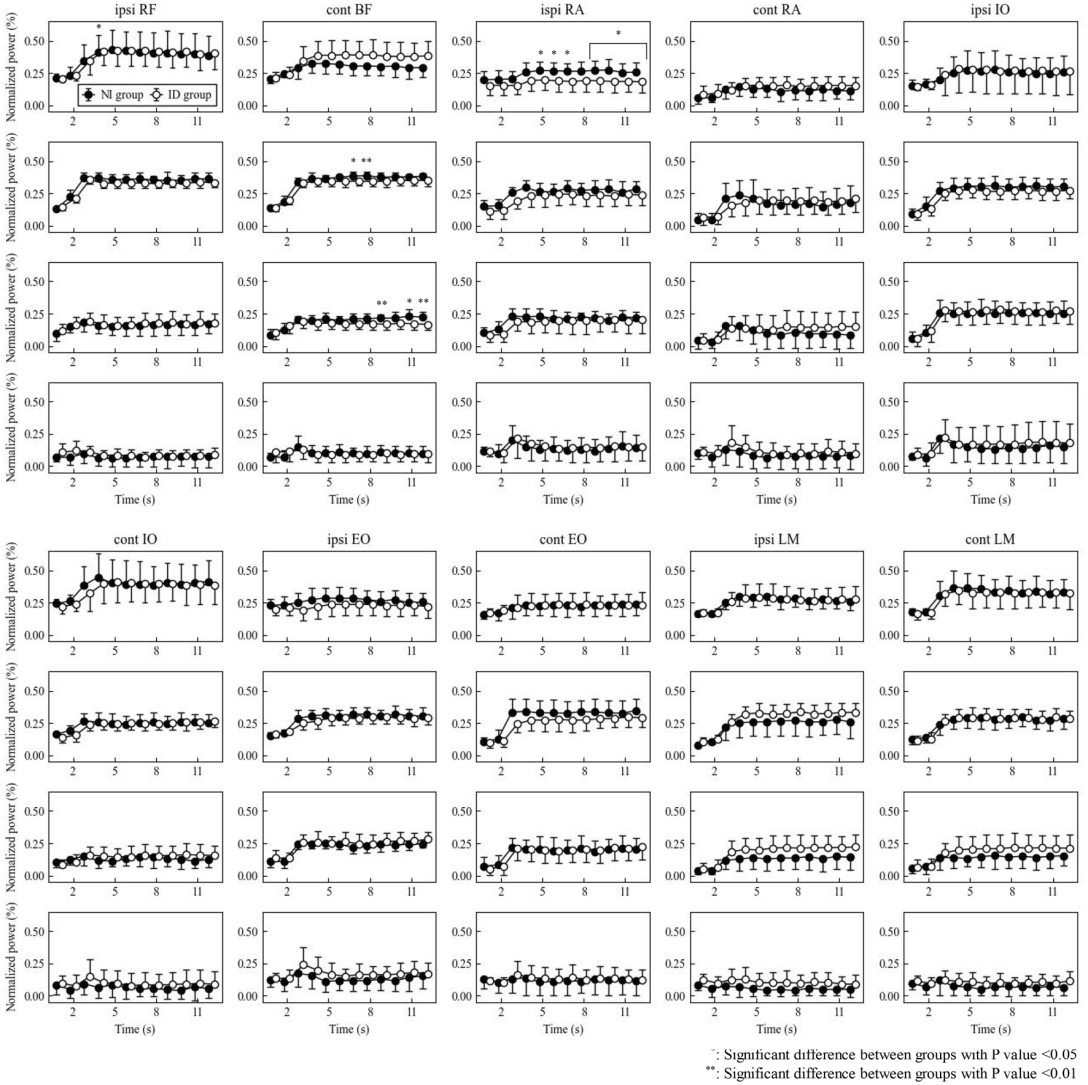
Power in each frequency band during dominant SLR. The HF, MF, LF and VLF band from top to bottom row in each muscle. The MF and LF bands of BF were significantly higher in the NI group (all P < 0.05, which of 0.0065 at 8 s in the MF band and 0.0080 and 0.0058 at 9 and 12 s in the LF band). The HF band of ipsilateral RA was significantly higher in the NI group at 5 to 7 and 9 to 12 s (all P < 0.05).

**Figure 4:**
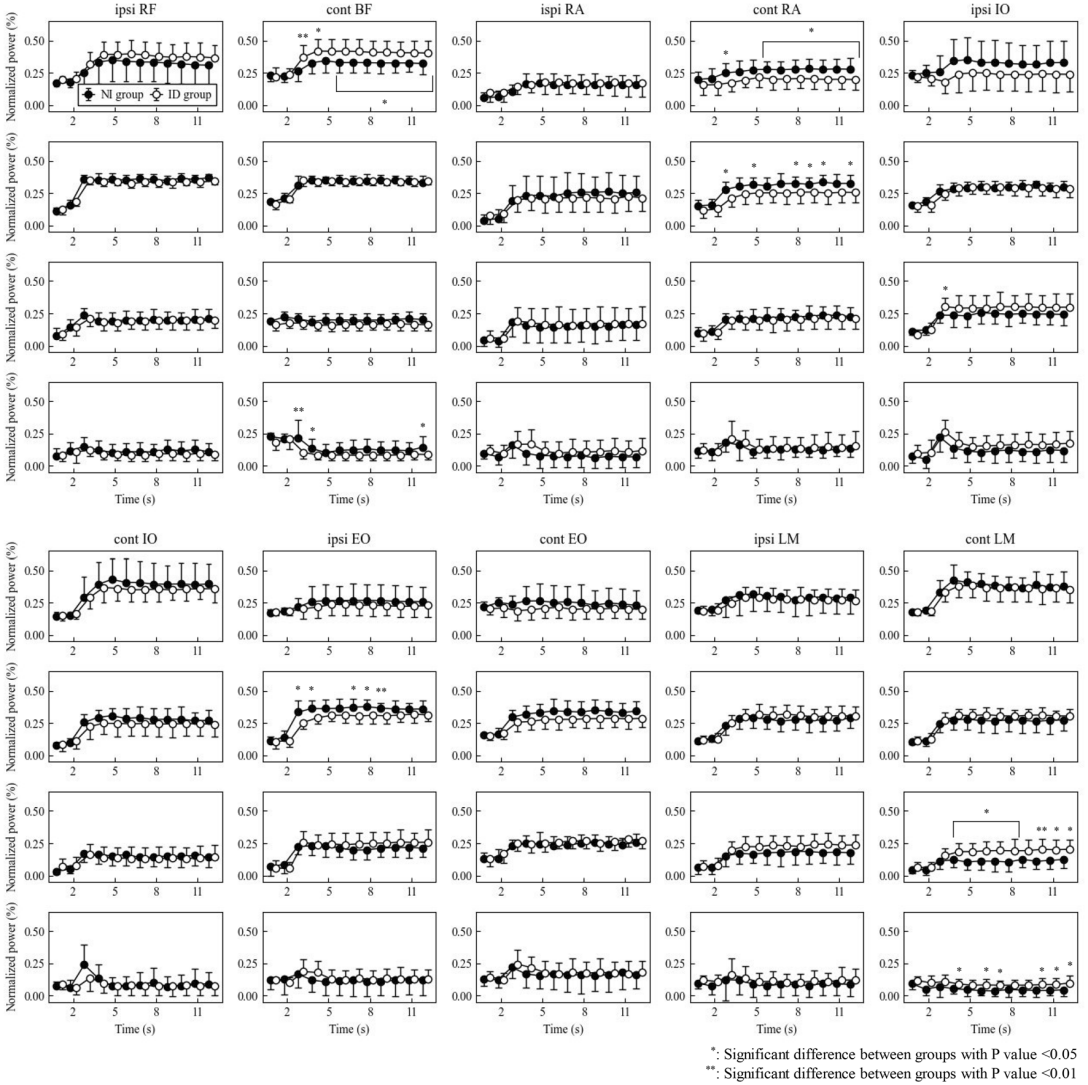
Power in each frequency band during nondominant SLR. The HF, MF, LF and VLF band from top to bottom row in each muscle. The HF band of BF was significantly higher in the ID group at almost all times during SLR (all P < 0.05, which of 0.0061 at 3 s), and the VLF band was higher in the NI group only at 3, 4 and 12 s (P = 0.0061, 0.0362 and 0.0365, respectively). The HF and MF bands of contralateral RA were significantly higher in the NI group at almost all times during SLR (all P < 0.05, which of 0.0076 at 10 s in the MF band). The LF band of ipsilateral IO was significantly higher in the ID group only at 3 s (P = 0.0272). The MF band of ipsilateral EO was higher in the NI group at almost all times during SLR (all P < 0.05, which of 0.0089 at 9 s).

Representative examples of scalograms of all 10 muscles during nondominant SLR are shown in Supplementary Fig. S2 online. The HF and MF bands were the largest on both sides, ranging from 0.3 to 0.4. The main results are as follows: For the dominant SLR, the MF and LF bands of BF showed significant interactions (P = 0.0094 and < 0.001, respectively), and the MF band was a significant between-group main effect (P = 0.0498). Both bands were significantly higher in the NI group than in the ID group (all P < 0.05, of which 0.0065 at 8 s in the MF band and 0.0080 and 0.0058 at 9 and 12 s in the LF band). There was also a significant between-group main effect for the HF band of ipsilateral rectus abdominis (RA) (P = 0.0274), which was significantly higher in the NI group than in the ID group at 5 to 7 and 9 to 12 s (P = 0.03028, 0.01974, 0.01384, 0.02784, 0.02443, 0.03942, 0.04227 and respectively).

For the nondominant SLR, the HF and VLF bands of BF were significant between-group main effects (P = 0.0350 and 0.0385, respectively) and interactions (P < 0.001 and 0.0028, respectively). The HF band was significantly higher in the ID group at almost all times during SLR (all P < 0.05, of which 0.0061 at 3 s), and the VLF band was higher in the NI group only at 3, 4 and 12 s (P = 0.0061, 0.0362 and 0.0365, respectively). The HF and MF bands of contralateral RA showed significant between-group main effects (P = 0.0315 and 0.0236, respectively), and both were significantly higher in the NI group than in the ID group at almost all times during SLR (all P < 0.05, of which 0.0076 at 10 s in the MF band). The LF band of ipsilateral IO showed a significant interaction (P = 0.0120) and was significantly higher in the ID group than in the NI group only at 3 s (P = 0.0272). The MF band of ipsilateral EO showed a significant between-group main effect (P = 0.0106) and was higher in the NI group at almost all times during SLR (all P < 0.05, of which 0.0089 at 9 s).

## Discussions

In this study, acceleration and EMG from the lower extremity and trunk were measured in patients with ICCI during SLR and compared according to their levels of walking independence. The results showed almost no group differences in acceleration, EMG onset, and RMS, and the EMG frequency analysis showed group differences in BF and some trunk muscles.

Previous studies reported that the percentage of walking independence decreases at age 70 or older^2^. Although no group differences were observed in this study, the mean age of the NI group exceeded 70 years old, which may become more pronounced with an increased sample size. It was also reported that a LEMS of 42 points or higher is associated with walking independence^3^. Significant group differences in LEMS were also observed in this study, with the NI group scoring 41 points on average below the cutoff value. However, there was no difference in the number of days of SLR acquisition, which may be attributed to the lower difficulty of the exercise, allowing SLR acquisition even with a lower LEMS. In addition, previous studies that measured the onset and RMS of EMG during SLR compared healthy subjects and patients with chronic pain^9,18–20^. Therefore, the lack of group differences in acceleration and the onset and RMS of EMG in this study may be attributed to the comparison being made for the same diseases and that these items change after the recovery phase rather than during the acute phase.

Conversely, when EMG was divided into four frequency bands by frequency analysis, significant group differences were found in BF and RA on the dominant side and in EO, in addition to these two muscles on the nondominant side. The HF band mainly reflects muscle fiber type 2 activity, whereas the MF band reflects the mixed activity of types 2 and 1^22^. In the present study, the ID group had higher HF bands in the BF, whereas the NI group had higher MF to VLF bands in the BF, HF and MF bands in the RA, and MF bands in the EO. This suggests that the ID group with high lower limb muscle strength mobilized the muscle fiber type 2 of the BF to stabilize the pelvis during SLR. In contrast, the NI group with low lower limb muscle strength may mobilized the type 1 of the BF or type 2 and 1 of the trunk muscles. This result was more pronounced in the nondominant SLR. The nondominant side was the axis leg for kicking the ball; therefore, coordination between the lower limb and trunk muscles was more important, and differences in walking independence might be more apparent when the nondominant side was exercised.

The results of this study underscore the significance of assessing muscle activity during exercises that engage multiple muscle groups, such as SLR, in addition to the conventional evaluation of LEMS during single-joint exercises for patients with ICCI. In future investigations, enhancing the intensity of SLR may reveal discernible differences in exercise speed and muscle activity. This can be achieved by attaching a weight to the lower limb on the exercise side, having patients perform SLR at maximum speed and increasing the raising angle to 30° or beyond. In clinical practice, it is imperative to consider that, even if an ICCI patient appears capable of SLR up to 30° of elevation at first glance, the activation method of the biceps femoris and trunk muscle groups responsible for stabilizing the pelvis may vary. Currently, detailed muscle activity can only be evaluated through electromyography. However, with the anticipated progress in future research, there is potential for the easy evaluation of muscle activity through visual examination and palpation in a clinical setting. Since SLR can be conveniently assessed in bed, it provides an opportunity to evaluate the extent of motor impairment and functional prognosis in patients, even in the acute phase when comprehensive assessment is hindered by rest limitations and pain.

This study presents several limitations. First, the sample size was small, particularly for the NI group. This may be because patients are more likely to achieve walking independence if they have sufficient muscle strength to perform SLR. Second, the level of walking independence was only assessed during discharge or transfer from our hospital. Therefore, it is necessary to evaluate the long-term prognosis after being discharged from the recovery hospital. Third, there was inadequate evaluation of muscle performance and strength. For example, the muscle activity patterns of the trunk and lower extremities prior to the injury were unknown, and some patients may have had some age-related changes before. Additionally, the muscle strength in the EMG-measured BF and trunk muscles were not clinically assessed. Fourth, although we considered the dominant and nondominant sides, some patients had left–right differences in LEMS, and this effect should be considered. Fifth, it cannot be definitively stated that there was no spinal shock effect at the time of SLR measurement. However, the interval between injury and measurement ranged from a minimum of 7 days to a maximum of 30 days. Additionally, all patients exhibited some muscle strength below the injured area, and in most cases, deep tendon reflexes were present. Based on these observations, we reasonably infer that the patients were likely beyond the spinal shock phase at the time of measurement. Finally, the large psoas muscle (iliopsoas), which is activated during SLR, was not evaluated^8^. Combined with ultrasound imaging, the EMG of the iliopsoas can be measured^23^, and adding this would provide additional results.

In conclusion, EMG frequency analysis of the lower limb and trunk muscles during SLR revealed that the ID group mobilized muscle fiber type 2 of the BF, and the NI group mobilized muscle fiber type 1 of the BF and types 2 and 1 of the trunk muscles to stabilize the pelvis. This result was more pronounced during nondominant SLR.

## Methods

### Ethical approval

All procedures were performed in accordance with the ethical standards of the institutional and national research committee and with the 1964 Helsinki Declaration and its later amendments or comparable ethical standards. This study was approved by the Medical Ethics Committee of the Japanese Red Cross Kobe Hospital (registry number: 200). All patients received both verbal and written explanations of the study, and written informed consent was obtained from each patient.

### Patients

**Figure 5:**
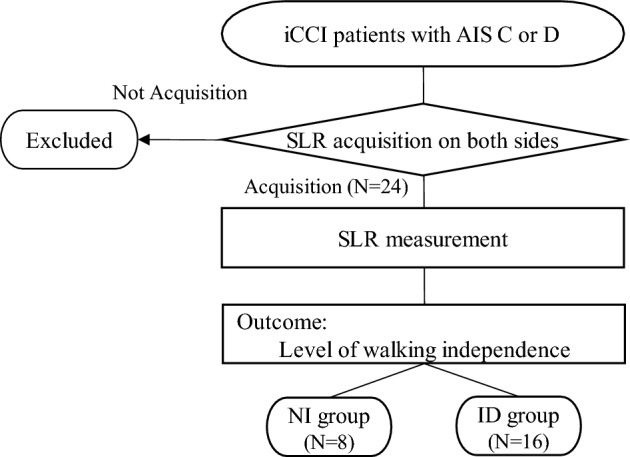
Flowchart from patient inclusion to outcome assessment.

A flowchart of this study is shown in Fig. 5. We included patients with ICCI whose ASIA Impairment Scale (AIS) grades were C or D. The inclusion criteria comprised patients with an AIS grade of C or D at the time of discharge or transfer from the hospital, those who were capable of independent walking and activities of daily living before the injury, and those with no discernible cognitive decline. Exclusion criteria encompassed complications such as lower extremity fractures that impeded measurement or a history of central nervous system disease.

After admission, patients were evaluated by SLR acquisition and the number of days until acquisition on each side. The criterion was the ability to raise their leg for 30° and hold it for 5 s^24^. We assessed patient’s LEMS as the comprehensive muscle strength of both lower extremities, either before or after the measurement. The level of walking independence upon discharge or transfer from our hospital was assessed on a scale of 0 to 8 points using item 12 (“Mobility Indoors”) of the SCIM. The nonindependent (NI) group was defined as those with a score of 3 or less who requires supervision or are unable to walk. The independent (ID) group were those with a score of 4 or more and can walk independently with or without aid^2,25^.

### Measurement procedures

Measurements were taken after the patients had undergone SLR on both sides. In the supine position with the legs straight on the treatment bed, the patients were instructed to raise each leg at 30° at normal speed without bending the knee and hold it for 10 s for three trials. They were shown the raising angle in advance and practiced it as needed. Measurements were first performed on the right leg and subsequently on the left leg. A rest period of at least 1 min was allowed between trials.

### Instruments

**Figure 6:**
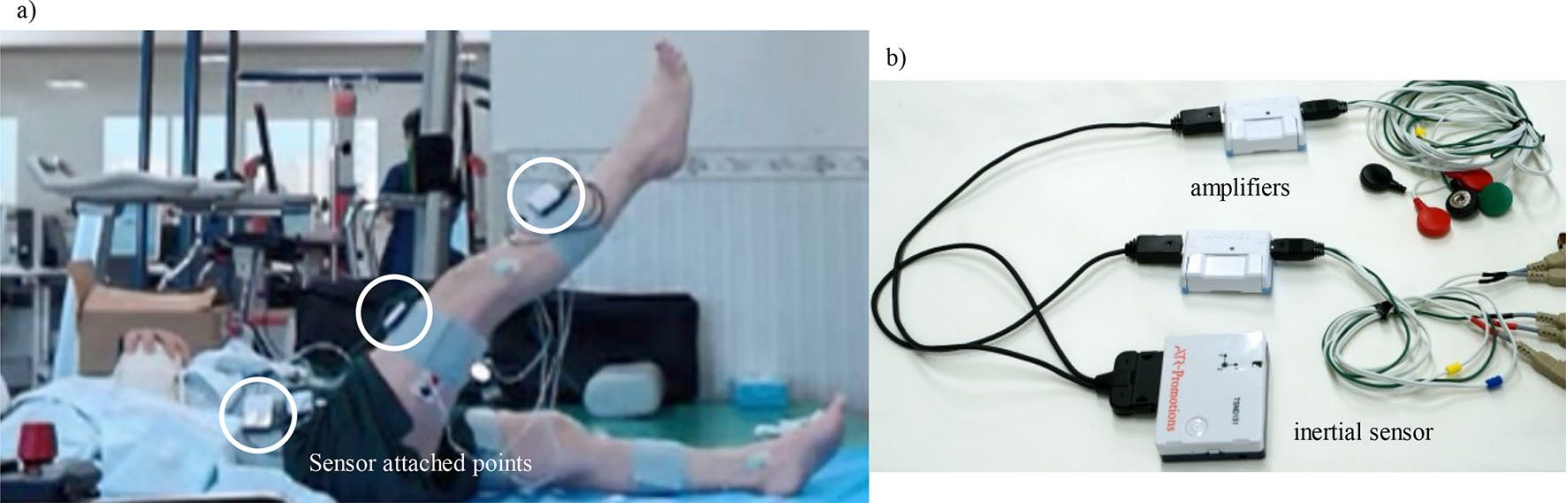
(**a**) SLR measurement scene and (**b**) instruments.

Three inertial sensors with built-in triaxial acceleration and angular velocity sensors (TSND151, ATR-promotions) were attached to the anterior superior iliac spine (ASIS), midthigh, and midlower leg on the exercise side. Pairs of disposable Ag/AgCl surface electrodes (SE-EXP-LEC60, Sekisui Kasei) with an interelectrode distance of 20 mm (center-to-center) were placed on 10 muscles: ipsilateral RF, contralateral BF, bilateral RA, IO, EO, and lumbar multifidus (LM)^26,27^. The skin was properly cleansed with ethanol before electrode placement. Data were measured synchronously at a sampling rate of 1000 Hz using amplifiers to measure biological signals (AMP-151, ATR-Promotions) and operated on software (ALTIMA, ATR-Promotions) using a laptop. Raw EMG signals processed using a bandpass filter with cutoff frequencies of 10 and 500 Hz (CMRR > 90 dB) were amplified and collected. The measurement setup and instruments are shown in Fig. 6.

### Data analysis

The SLR start point was identified from the angular velocity (pitch angle) around the Y-axis (mediolateral direction) of the inertial sensor attached to the lower leg. Specifically, the slope of the moving average for each sample was computed, and the point at which one-eighth of the maximum slope was surpassed by 50 consecutive points was identified. We analyzed the data for 12 s (2 s before and 10 s after the SLR start point).

**Figure 7:**
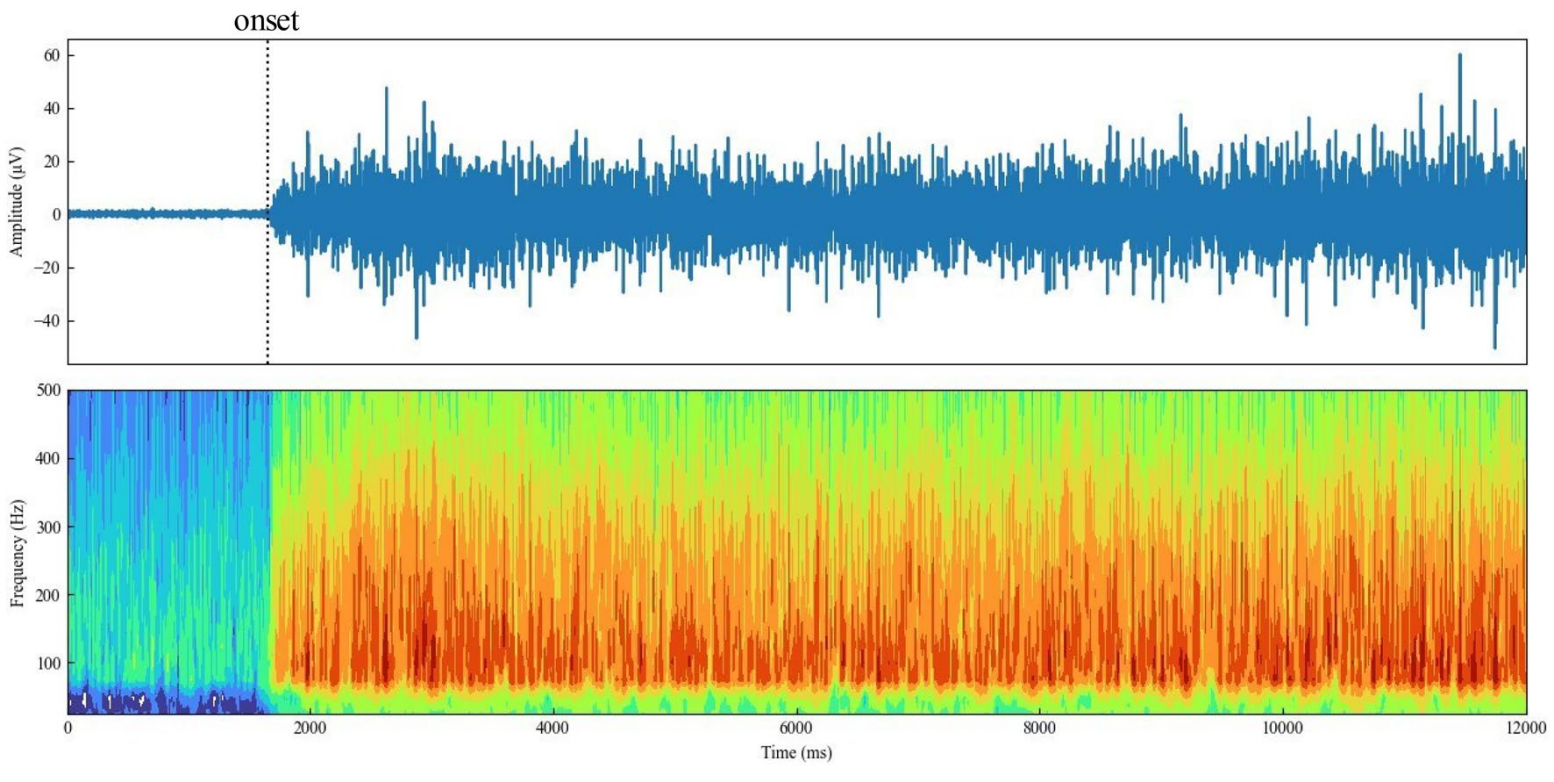
Example of EMG (top row), scalogram (bottom row) and muscle activity onset of ipsilateral RF. To enhance the readability of the scalograms, values were transformed using the natural logarithm, and the range of the color bars was adjusted.

From the acceleration, the RMS of the amplitude every 50 ms for each axis and the combined acceleration were calculated using a 1-Hz high-pass filter to remove the gravitational component. From the EMG, the RMS of the amplitude every 50 ms and the onset time were calculated using a 65-Hz high-pass filter to remove the electrocardiographic component. The RMS for each muscle was normalized to a maximum value of 1. The onset time was calculated using the total power, i.e., the sum of the squared amplitudes below 500 Hz extracted by frequency analysis using continuous wavelet transform (CWT). The onset was defined as 30 consecutive points from the SLR start point to 2 s later, in which the total power exceeded the resting mean plus three times the standard deviation, expressed as the difference from the SLR start point. Figure 7 shows an example of the EMG analysis of the ipsilateral RF.

Next, the CWT was performed on the raw EMG with 20 scales, and the power in the frequency bands was calculated every 25 Hz. For each frequency band, the average value for 1 s at rest was subtracted and values less than 0 were replaced with 0. The power was added to calculate the very-low-frequency (VLF; from 0 to 25 Hz), low-frequency (LF; from 25 to 50 Hz), medium-frequency (MF; from 50 to 100 Hz), and high-frequency bands (HF; 100 Hz or higher)^22,28–31^. Finally, the power for each time point was normalized to a total power of 1.

We used complex Morlet wavelets as the mother wavelet and set the bandwidth parameter and center frequency to 1 Hz based on a prior study^32^. These analyses were performed using Python and PyWavelets, i.e. an open-source wavelet transform module in Python.

### Statistical analysis

Statistical analysis was performed separately for the patient's dominant and nondominant sides. The dominant leg was determined by the patient's self-report in response to the question of which leg he used to kick the ball. Variables, excluding the onset time, were averaged every second, and the average of three trials was used.

Fisher’s exact test was used to compare gender ratios between groups. Regarding the other basic information and onset time, an unpaired *t* test or Mann–Whitney *U*-test was used after checking the normal distribution and homogeneity of variance of each data point for group comparison. The other acceleration and EMG variables were analyzed using a split-plot factorial design for linear mixed models, including two factors: between groups as the noncorrespondence factor and time as the repeated-measures factor. Post-hoc tests were performed when a significant main effect between the groups or interaction was observed.

Statistical analyses were performed with a two-tailed test using Modified R Commander 4.2.2^33^. The level of statistical significance was set at a P value < 0.05. Descriptive statistics (mean and standard deviation) were used to summarize the results.

### Supplementary Information

Supplementary Information.

## Data Availability

The datasets analyzed during the current study are available from the corresponding author on reasonable request.
