# Supplementary material for: Effects of the walking independence on lower extremity and trunk muscle activity during straight-leg raising following incomplete cervical cord injury
Source: Sci Rep. 2024 Feb 22;14:4363. doi: 10.1038/s41598-024-55039-z (PMC10883988; doi:10.1038/s41598-024-55039-z)
Supplement: Supplementary file 1 — Supplementary Information. [file 41598_2024_55039_MOESM1_ESM.pdf]

Title:

Effects of the walking independence on lower extremity and trunk muscle activity during straight-leg raising following incomplete cervical cord injury

Authors:

[1] Tatsuya Sugimoto * [1,2]

[2] Ryoto Yoshikura [3]

[3] Toshiyuki Maezawa [1]

[4] Kojiro Mekata [4]

[5] Yuya Ueda [5]

[6] Hiroshi Kawaguchi [3]

[7] Shintaro Izumi [3,6]

	NI (n=8)	ID (n=16)	P values
modified Frankel classification			
initial evaluation (from A to E)	C1 1, C2 5, D0 2	C2 1, D0 8, D1 3, D2 3, D3 1	**0.015**
SLR measurement	C2 8	D0 2, D1 6, D2 5, D3 3	**<0.001**
discharge/transfer	C2 8	D1 6, D2 7, D3 3	**<0.001**
SCIM score for "Mobility for Moderate Distances (10-100 meters)"			
initial evaluation (from 0 to 8 points)	0 (0)	4.0 (3.6)	None
SLR measurement	0.8 (1.4)	6.4 (2.2)	**<0.001**
discharge/transfer	1.5 (1.6)	7.5 (1.2)	**<0.001**
Walking Index for Spinal Cord Injury II			
initial evaluation (from 0 to 20 points)	0.5 (1.4)	12.7 (9.1)	**0.0043**
SLR measurement	6.9 (6.5)	18.4 (2.8)	**<0.001**
discharge/transfer	10.4 (7.9)	19.9 (0.25)	**<0.001**

Table S1. Other walking indicators for each group. Data are shown as mean (standard deviation). P values less than 0.05 are highlighted in bold.

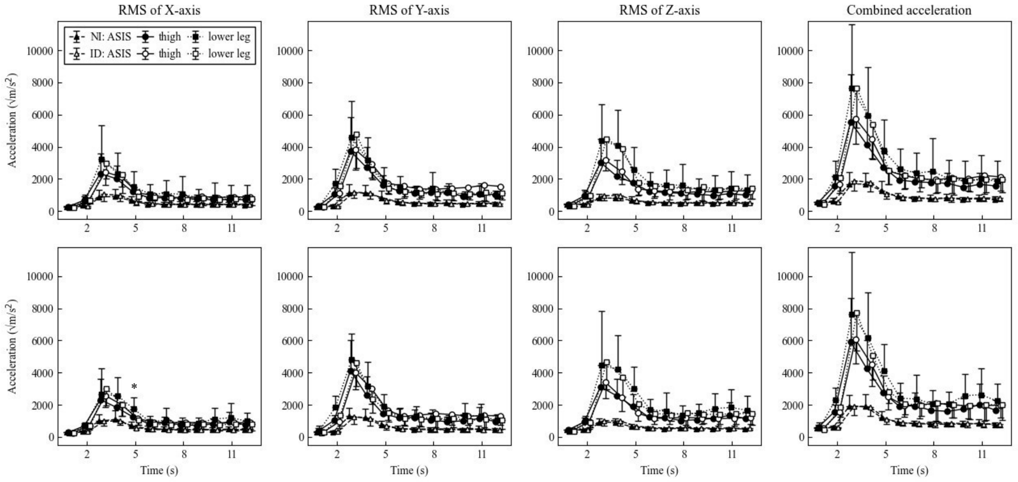

*: Significant difference between groups with P value <0.05

Figure S1. Acceleration RMS and combined acceleration during dominant (top row) and nondominant (bottom row) SLR. The RMS of the X-axis in the lower leg during the nondominant SLR was significantly higher in the NI group at 5 seconds (P=0.0048).

SLR side	muscles	NI (n=8)	ID (n=16)	P values
Dominant	ipsi RF	-334.7 (131.2)	-290.5 (93.3)	0.41273
	cont BF	-174.9 (190.9)	-191.9 (296.6)	0.48958
	ipsi RA	332.3 (273.8)	469.9 (399.7)	0.40631
	cont RA	461.4 (356.3)	425.4 (343.1)	0.83324
	ipsi IO	-2.7 (359.2)	7.0 (252.5)	0.94658
	cont IO	407.7 (397.4)	383.6 (491.5)	0.90524
	ipsi EO	421.5 (368.1)	157.9 (167.8)	0.08835
	cont EO	416.7 (483.8)	215.4 (298.4)	0.31355
	ipsi LM	536.7 (402.9)	489.5 (499.5)	0.95882
	cont LM	453.1 (604.0)	474.1 (395.0)	0.93515
Nondominant	ipsi RF	-311.6 (68.7)	-239.7 (131.5)	0.09221
	cont BF	-88.0 (307.5)	-258.6 (112.1)	0.21439
	ipsi RA	253.1 (473.5)	331.2 (395.3)	0.48542
	cont RA	411.4 (389.1)	385.8 (365.8)	0.88442
	ipsi IO	316.1 (480.7)	70.2 (275.0)	0.21147
	cont IO	120.1 (170.9)	496.0 (472.8)	**0.01521**
	ipsi EO	165.4 (408.5)	336.8 (393.5)	0.34835
	cont EO	180.0 (312.1)	132.9 (260.9)	0.92034
	ipsi LM	595.8 (595.2)	651.9 (564.9)	0.82979
	cont LM	626.8 (521.6)	395.9 (486.5)	0.34112

Table S2. Muscle activity onset time (ipsi: ipsilateral, cont: contralateral). The onset times for the RF and BF were calculated for all trials. For the trunk muscles, patients whose onset times could not be calculated in all three trials

per leg were excluded. Data are shown as mean (standard deviation). P values less than 0.05 are highlighted in bold.

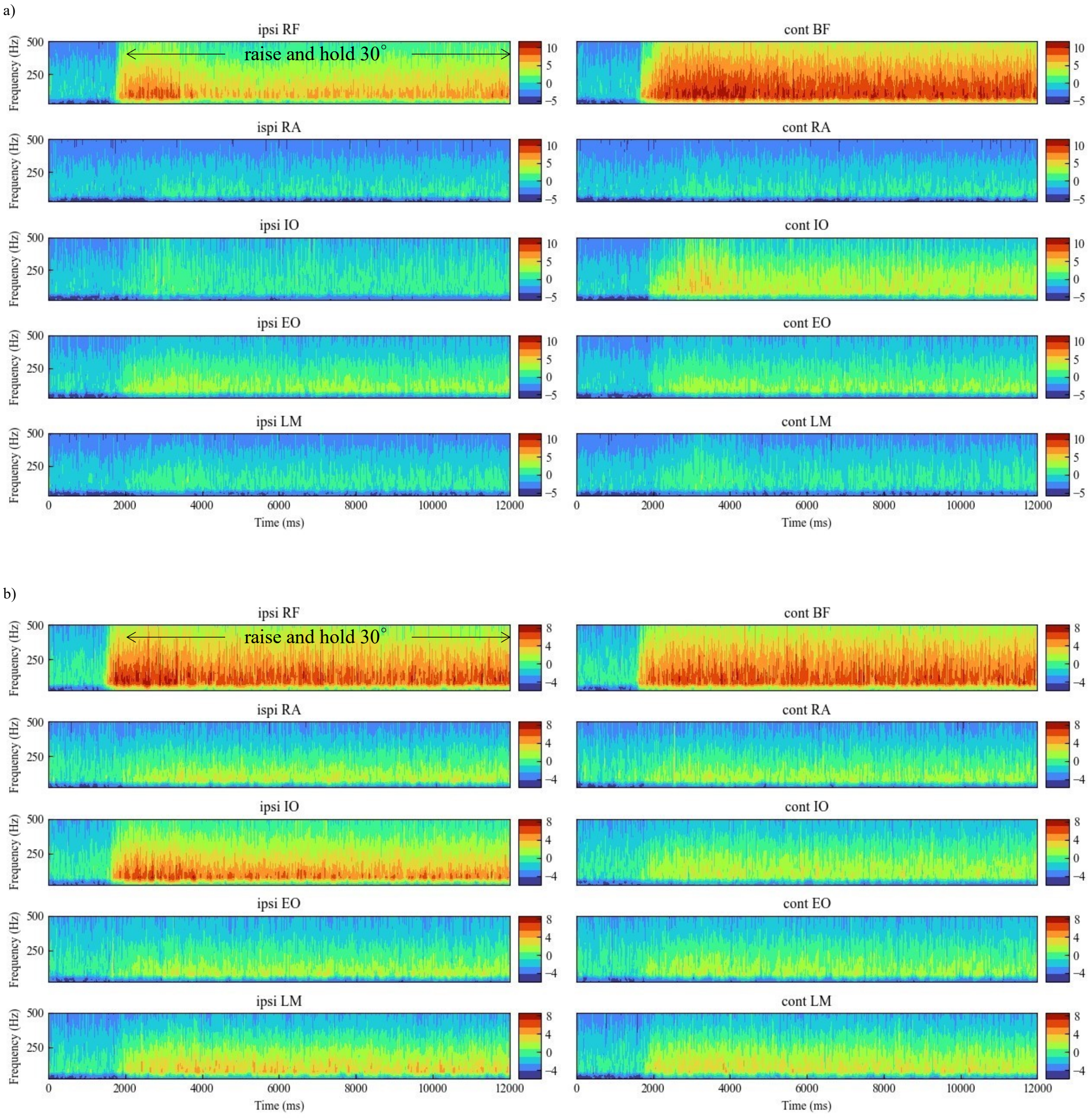

Figure S2. Example of scalograms of all 10 muscles during nondominant SLR, one each from the NI group (a) and ID group (b). To enhance readability, all values were transformed using the natural logarithm, and the range of the color bars was adjusted.
